# Comparison of the Skeletal and Dento-Alveolar Changes Obtained with a Customized Elastodontic Appliance and Twin Block: A Prospective Investigation

**DOI:** 10.3390/children12091147

**Published:** 2025-08-28

**Authors:** Valentina Lanteri, Andrea Abate, Margherita Donelli, Cinzia Maspero, Enrica Tessore, Maria Elena Grecolini, Francesca Olivi, Matilde Dalmazzini, Alessandro Ugolini

**Affiliations:** 1 Surgical, Medical and Dental Department, University of Modena and Reggio Emilia, 41124 Modena, Italy; valentina.lanteri@unimi.it (V.L.); enricatessore@tin.it (E.T.); dott.elena@grecoliniortodonzia.it (M.E.G.); f.olivimocenigo@gmail.com (F.O.); dmatilde23@gmail.com (M.D.); 2 Department of Sciences Integrated Surgical and Diagnostic, University of Genova, 16132 Genova, Italy; alessandro.ugolini@unige.it; 3 Department of Biomedical Surgical and Dental Sciences, University of Milan, 20142 Milan, Italy; margherita.donelli@unimi.it (M.D.); cinzia.maspero@unimi.it (C.M.); 4 Fondazione IRCCS Cà Granda, Ospedale Maggiore Policlinico, 20142 Milan, Italy

**Keywords:** twin block, elastodontic appliance, cephalometric analysis, growing patients

## Abstract

Objectives: This study aimed to compare the skeletal and dentoalveolar effects of a fully customized elastodontic appliance with those of the traditional Twin Block appliance in growing patients with Class II malocclusion during the mixed dentition phase. Methods: A total of 35 patients were included: 18 treated with a customized elastodontic appliance (C-Ela group) and 17 with a Twin Block appliance (TB group). Digital dental models and lateral cephalometric radiographs were obtained at baseline (T1) and after 12 months of treatment (T2). All patients were treated by experienced clinicians according to standardized appliance protocols. Data analysis was performed by a blinded operator using Ortho Analyzer and Dolphin Imaging software. The Shapiro–Wilk test was applied to verify the normal distribution of the data. Paired-sample *t*-tests were used to assess within-group changes between T1 and T2. For intergroup comparisons two-tail independent-sample *t*-tests were used, and chi-square tests were used for categorical variables. Statistical significance was set at *p* < 0.05. Results: Both groups showed significant intragroup improvements in overjet (C-Ela: −2.77 ± 2.07; TB: −2.30 ± 2.72 mm), overbite (C-Ela: −1.79 ± 1.95; TB: −1.40 ± 2.65 mm), and sagittal molar relationship (*p* < 0.05) after treatment. The C-Ela group exhibited a significantly greater reduction in anterior dental crowding (*p* < 0.05) and better control of upper (C-Ela: −4.93 ± 7.65°; TB: −1.80 ± 5.72°) and lower incisor inclination (C-Ela: +1.70 ± 4.80°; TB: +4.35 ± 6.22°). In intergroup comparisons, the TB group showed a significantly greater proclination of the lower incisors at T2 (L1/Go-Gn: +4.35°; L1/A-Pog: +1.44 mm), whereas the C-Ela more effectively limited these changes (L1/Go-Gn: +1.70°; L1/A-Pog: +1.18 mm). Skeletal analysis revealed an increase in ANB angle in both groups (C-Ela: −1.49 ± 2.62°; TB: −1.78 ± 2.78°), with no statistically significant intergroup differences, and no other skeletal parameters showed significant between-group changes. Conclusions: Both appliances effectively corrected Class II malocclusions. However, the customized elastodontic device provided better dentoalveolar control, particularly in managing anterior crowding and incisor inclination. Its individualized fit may enhance biomechanical precision and improve overall treatment outcomes in growing patients.

## 1. Introduction

The timing of orthodontic treatment has long been a debated topic, especially regarding the benefits of early intervention to address skeletal discrepancies and dental crowding. Studies show that malocclusions, such as Class II anomalies, often worsen with age, making their correction more challenging in later stages [1,2,3]. Early treatment during mixed dentition not only prevents further complications but also simplifies subsequent orthodontic care [4,5]. Class II malocclusion, one of the most common orthodontic issues, is typically characterized by mandibular retrusion, with approximately 70% of patients showing a normally developed maxilla but an underdeveloped mandible. Given its multifactorial etiology, including genetic and environmental factors, Class II malocclusion requires comprehensive diagnostic and therapeutic approaches [6,7].

The Twin Block appliance, introduced by Clark in 1982, has become a cornerstone for correcting Class II malocclusions [8]. Unlike other functional appliances, its design allows for mandibular advancement through acrylic resin blocks that slide on a 70° incline, promoting mandibular protrusion. This appliance is unique in its two-phase protocol: the active phase, where the malocclusion is corrected, and the passive phase, during which a retention device stabilizes the results over time. The appliance is designed for continuous wear, which helps maintain the desired mandibular posture. Its efficacy has been demonstrated in achieving dento-skeletal corrections by stimulating mandibular growth and improving neuromuscular balance, making it particularly effective during the pubertal growth spurt [9].

The mandibular advancement it promotes depends heavily on the patient’s biological response, specifically the adaptability of the condylar cartilage to growth stimuli [10].

Prefabricated elastodontic devices such as the Eruption Guidance Appliance (EGA) have shown success in early mixed dentition, particularly as an interceptive option for less severe Class II cases [4]. EGAs guide dental eruption and apply light elastic forces to improve mandibular growth and overjet correction [11]. They have been proven effective in reducing overjet, resolving anterior crowding, and improving molar relationships, with stable outcomes maintained into the permanent dentition [5,12,13,14]. However, their reliance on preformed designs limits adaptability, making them less versatile than the Twin Block for addressing complex skeletal discrepancies [11].

Recent advances in digital orthodontics have further expanded the potential of eruption guidance appliances and elastodontic devices. In fact, customization through 3D imaging and printing allows for the precise tailoring of these devices to individual anatomical needs, enhancing treatment predictability and patient outcomes. Digital workflows eliminate human error associated with prefabricated designs, providing a more targeted approach to skeletal and dentoalveolar corrections.

Despite their widespread use, to the best of the authors’ knowledge, no studies have directly compared fully customized elastodontic appliances with traditional functional devices such as the Twin Block. Emerging evidence indicates that elastodontic appliances can enhance cephalometric and dentoalveolar parameters, potentially contributing to the correction of skeletal and dental relationships [15]. Nevertheless, the variability of results and the lack of direct comparative data with conventional appliances highlight the need for further research in this field.

Therefore, the present study aims to address this gap by comparing the skeletal and dentoalveolar effects of customized elastodontic appliances with those of the Twin Block appliance in the treatment of Class II malocclusion during the mixed dentition phase.

## 2. Materials and Methods

This longitudinal prospective analysis focused on the skeletal and dentoalveolar changes observed after the use of both customized and preformed Twin Block appliances. The research was conducted at the Department of Biomedical, Surgical and Dental Sciences of the University of Milan, Fondazione IRCCS Ca’ Granda, Ospedale Maggiore Policlinico, between June 2022 and December 2024. The study protocol was approved by the competent Institutional Review Board (IRB) as part of the Research Protocol of Fondazione IRCCS Cà Granda Ospedale Maggiore Policlinico. Operative Unit 420, Current Research N. 1, year 2022. The research adhered to the principles outlined in the World Medical Association Declaration of Helsinki. Informed consent was obtained from the parents of all participants, authorizing the use of their diagnostic records for research purposes.

### 2.1. Subjects

Eligible records, both for patients treated with Twin Block appliances and customized elastodontic devices, were randomly selected using a digital randomization tool, which generated a computer-based random sequence. This approach was applied to ensure an unbiased allocation of records and to minimize selection bias.

The inclusion criteria for subject selection were:

Digital dental models and lateral cephalograms were obtained at two time points: before treatment (T1) and immediately after a 12-month treatment period (T2). The 12-month interval between T1 and T2 was not arbitrarily chosen but reflects the standard active treatment duration for functional appliances, as consistently reported in the literature. Several recent studies have adopted a similar timeframe for evaluating treatment effects, including Lombardo et al. (2024) [16], Gülsoy & Yavan (2023) [17], and Xu et al. (2024) [18].

The inclusion criteria for this study required patients to have (1) an overjet greater than 4 mm, (2) bilateral molar relationships either in full Class II or end-to-end, (3) ANB angle exceeding 4°, (4) noticeable enhancement of the facial profile upon forward posturing of the mandible, (5) cervical vertebral maturation stage 3 (CVM) at the initial time point (T1), (6) no prior orthodontic treatment apart from maxillary expansion. Exclusion criteria were as follows: (1) Angle Class III malocclusion; (2) the presence of posterior or anterior crossbites; (3) retroclined upper incisors; (4) craniofacial syndromes; and (5) signs of temporomandibular dysfunction.

Eighteen children who met the inclusion criteria were treated with customized elastodontic appliances (C-Ela Group), with a mean age of 11.1 ± 0.7 years (6 males, 12 females). Additionally, seventeen children treated with Twin Block appliances (TB Group) and meeting the same inclusion criteria were selected, with a mean age of 11.7 ± 0.5 years (5 males, 12 females).

Treatment was carried out by two orthodontists with equivalent backgrounds in managing functional appliance therapy, as reflected by their similar years of practice and the number of cases previously treated.

### 2.2. Treatment Protocol

All individuals in the C-Ela group underwent therapy with a customized elastodontic appliance (Digital Service Leone, Sesto Fiorentino, FI, Italy). Each device was fabricated based on a specific prescription form, which included digital dental models, extraoral and intraoral photographs, and a lateral cephalometric radiograph.

The construction bite was tailored to each patient’s clinical needs, allowing for mandibular advancement aimed at correcting Class II malocclusion [6,19]. The mandibular position was progressively adjusted in 2.5 mm increments until an incisal edge-to-edge relationship was achieved. The design also incorporated analysis of the Bolton ratio to ensure proper arch coordination, and the Ballard-Wyle and Moyers indices to estimate the mesiodistal width of unerupted permanent teeth [20,21].

Specific construction features [6,19] were included to address overbite correction, such as anterior intrusion and posterior extrusion. Clinical instructions for resolving anterior crowding, overbite reduction, and midline deviations were integrated into the virtual setup. Where indicated, overcorrection of the midline was planned.

**Figure 1:**
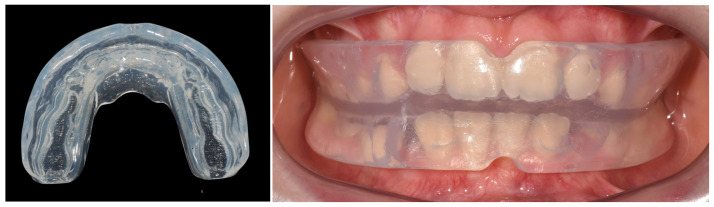
Extraoral and intraoral views of the customized elastodontic appliance at the time of delivery. The (**left image**) shows the extraoral morphology of the elastomer device, designed to guide dental eruption and modulate occlusal forces. The (**right image**) illustrates the intraoral positioning upon insertion, highlighting its adaptation to the dental arches.

Upon clinician approval of the digital setup, the customized elastodontic appliance was created using 3D printing. All appliances were fabricated from a biocompatible polymer–elastomer blend, chosen for its rigidity and patient comfort (Figure 1).

Each device shared a consistent design, with two flanges—one vestibular and one lingual. The vestibular flange acted as a lip bumper to reduce perioral muscle hyperactivity and discourage mouth breathing, especially by mitigating the excessive tone of the orbicularis oris and lower lip muscles. The lingual flange featured a ramp that promoted proper tongue posture against the palatal spot and prevented interposition or low tongue position. This ramp also encouraged physiological swallowing and supported the benefits of concurrent myofunctional or speech therapy when prescribed.

Between the flanges, integrated eruption guides directed the eruption of permanent teeth, contributing to the control of overjet and overbite and facilitating midline correction. All patients received standardized instructions for appliance use, following the same clinical protocols and guidelines previously described in the literature [5,22]. Each subject was instructed to wear the device nightly, along with an additional 2 h of daytime use per day for a period of one year. The daytime wear could be divided into multiple sessions, provided that each lasted at least 30 min. Daily use of both eruption guidance appliances was to be combined with specific myofunctional exercises, including intermittent clenching and maintaining lip contact.

**Figure 2:**
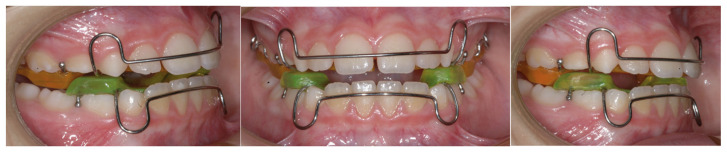
Intraoral view of the Twin Block appliance at the time of delivery. The image shows the device in position, illustrating the occlusal relationship and the acrylic bite blocks designed to advance the mandible and promote functional jaw adaptation.

The TB group was treated using a Twin Block appliance designed in accordance with Clark’s original concept (Figure 2).

The device consisted of separate maxillary and mandibular plates that adapted to the dentition, alveolar processes, and surrounding support structures. Retention was achieved using Delta or Adams clasps on the first permanent molars bilaterally, while anterior retention was provided by 0.030-inch ball clasps or arrow clasps placed interproximally. The specific configuration of the clasps was customized based on each patient’s dental development at the time of appliance fabrication. In the lower arch, interproximal ball hooks were generally positioned between canines and incisors, as recommended by Clark [23].

Bite registration for appliance construction was taken with the incisors in an edge-to-edge position if the initial overjet measured 7–8 mm. For patients presenting with an overjet greater than 8 mm, a staged advancement protocol was adopted. The first registration placed the mandible halfway between centric relation and edge-to-edge, followed by a second activation into a full edge-to-edge position approximately 3–4 months later. Vertical opening of 5–7 mm in the posterior region was incorporated into the construction bite to accommodate the posterior bite blocks.

The Twin Block system is able to control vertical development through selective reduction of the acrylic base. In patients with a hypodivergent growth pattern, reduced lower anterior facial height, or a pronounced curve of Spee, the posterior acrylic of the upper bite block was selectively trimmed to promote eruption of the lower molars and premolars.

All patients were instructed to wear the appliance for at least 22 h per day, removing it only during meals and sports activities, until completion of the functional phase.

### 2.3. Records Examination and Data Collection

Digital models were obtained for all patients at baseline (T1) and after one year of treatment (T2) in centric relation using an intraoral scanner (Trios 3, 3Shape D250 laser, 3Shape, Copenhagen, Denmark). All digital data sets from both groups were evaluated in random order by a single blinded examiner (A.A.), who carried out all measurements using the 3Shape Ortho Analyzer software (version 1.7, https://www.3shape.com/en/scanners/trios-3-basic (accessed on 3 March 2024)).

The following measurements were assessed on the digital dental casts:

1. Overjet (mm): horizontal distance between the incisal edges of the maxillary and mandibular central incisors.
2. Overbite (mm): vertical distance between the same incisal edges.
3. Anterior crowding: Based on Little’s Irregularity Index [24], the sample was divided into two categories: normal crowding (0–3 mm) and a combined “moderate/severe crowding” group, which included both moderate crowding (4–6 mm) and severe crowding (>6 mm), defined as crowding greater than 3 mm.
4. The arch was classified as crowded in the presence of overlapping incisors more than 3 mm; otherwise, it was recorded as normally aligned.
5. Angle’s classification:
  - Class I was defined when the mesiobuccally cusp of the upper first molar occluded within 2 mm of the buccal groove of the lower first molar.
  - Class II was assigned when this distance exceeded 2 mm.

Canine relationships were determined by measuring the distance from the tip of the maxillary canine to the contact point between the mandibular canine and the first deciduous molar:

  - Class I was defined as ≤1 mm;
  - Class II as >1 mm.

Subjects were also categorized as Class I/II when a unilateral Class II molar relationship was present.

**Figure 3:**
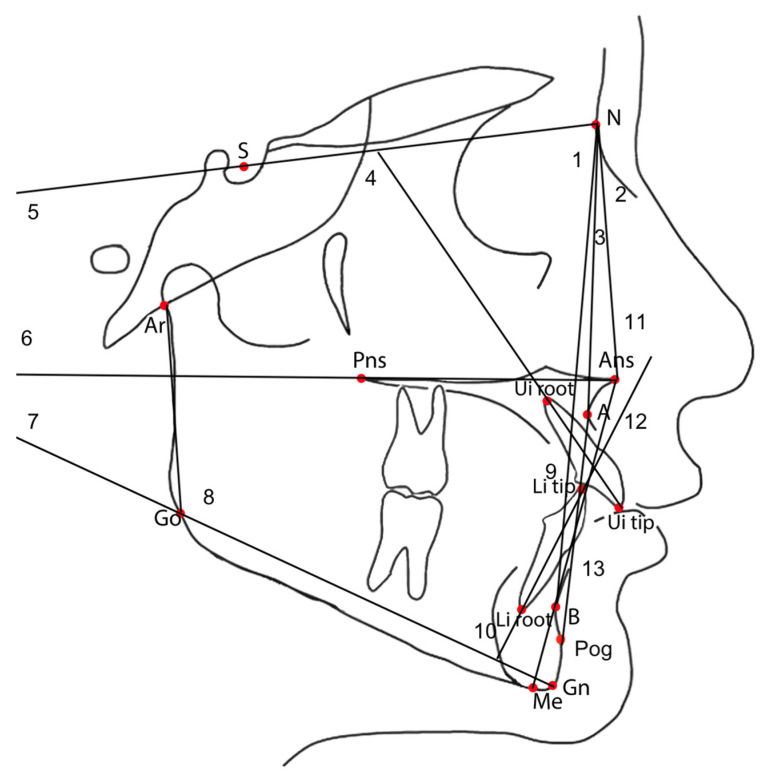
Linear and angular cephalometric parameters assessed in this study included the following: (1) SNA: the angle formed between the S-N and N-A lines; (2) SNB: the angle between the S-N and N-B lines; (3) ANB: the angular difference between the N-A and N-B lines; (4) U1-SN: the angle between the long axis of the maxillary central incisor and the S-N line; (5) SN/Go-Gn: the intersection angle between S-N and mandibular plane Go-Gn; (6) SN/Ans-Pns: the angle between the cranial base (S-N) and the palatal plane (Ans-Pns); (7) Ans-Pns/Go-Gn: the angle between the palatal and mandibular planes; (8) Ar-Go-Me: the total gonial angle, defined by the intersection of lines Ar-Go and Go-Me; (9) U1-L1: the interincisal angle, formed by the axes of upper and lower central incisors; (10) L1-Go-Me: the angle between the mandibular incisor’s long axis and the mandibular plane (Go-Gn); (11) N-Ans: measurement of the upper anterior vertical facial height; (12) Ans-Me: measurement of the lower anterior vertical facial height; (13) L1/A-Pog: the sagittal position of the lower incisor in relation to the A-Pog line.

Lateral cephalometric radiographs were acquired at T1 and T2 using the same digital unit (Sirona^®^ Dental Systems GmbH, 64625 Bensheim, Germany) with a constant focus-to-receptor distance of 150 cm (Figure 3). Cephalometric tracings were performed by a second blinded operator (A.U.) using Dolphin Imaging software version 5.0 (Dolphin Imaging and Management Solutions; Los Angeles, CA, USA, https://www.dolphinimaging.com (accessed on 1 September 2024)). A total of 15 anatomical landmarks were used to analyze skeletal and dentoalveolar changes [25,26].

### 2.4. Statistical Analysis

Sample size estimation was performed using the G*Power software (version 3.1.9; available at https://www.psychologie.hhu.de/arbeitsgruppen/allgemeine-psychologie-und-arbeitspsychologie/gpower (accessed on 7 July 2025)). The sample size adopted in the present study was based on the calculation reported by Lombardo et al. [16], a 1.7° difference in ANB with a standard deviation of 1.4° (Cohen’s *d* = 1.21) was assumed, resulting in a required minimum of 15 subjects per group (α = 0.05; power = 0.80).

Statistical analysis was conducted using SPSS software (version 25.00; IBM Corp., Armonk, NY, USA; https://www.ibm.com/support/pages/release-notes-ibm®-spss®-statistics-250 (accessed on 2 July 2024)). Changes in cephalometric and dentoalveolar parameters—such as overjet, overbite, sagittal molar relationship, and anterior dental crowding—between T1 and T2 were assessed and compared between the two study groups.

The Shapiro–Wilk test was applied to evaluate the normality of the data distribution. As the variables followed a Gaussian distribution, parametric tests were used, and results were expressed as means and standard deviations (SD). Comparison of baseline demographic and clinical characteristics between groups was performed using the independent samples *t*-test and chi-square test, as appropriate.

Intragroup differences from T1 to T2 were analyzed with the paired *t*-test for all cephalometric parameters. Differences in the distribution of sagittal molar and canine relationships, as well as anterior crowding, between groups at both time points were evaluated using the chi-square test. Intergroup comparisons of cephalometric changes after treatment with the two different appliances were carried out using the independent samples *t*-test.

A *p*-value < 0.05 was considered statistically significant.

Data collection was performed by the principal investigator (A.A.), with all records anonymized. To assess reproducibility, 10 randomly selected digital models and cephalograms were re-evaluated after 15 days by a second examiner (L.V.) and then reanalyzed by the first. Intra- and inter-operator reliability showed high agreement (ICC > 0.93), and method error, assessed using Dahlberg’s formula, was minimal (0.21 mm for linear and 0.35° for angular measurements).

## 3. Results

Demographic and clinical characteristics at baseline are reported in Table 1. No statistically significant differences were found between groups for any variable, as confirmed by independent sample *t*-tests and chi-square tests.

Similarly, skeletal and dentoalveolar parameters showed no significant intergroup differences at T1 (Table 2).

Both groups showed significant reductions in overjet and overbite from T1 to T2. In the C-Ela Group, overjet decreased from 5.25 mm to 2.48 mm and overbite from 3.99 mm to 2.2 mm; in the TB Group, overjet reduced from 4.93 mm to 2.63 mm and overbite from 3.63 mm to 2.63 mm (Table 3). Intergroup comparison revealed no significant difference in overjet and overbite reduction.

Chi-square analysis of molar and canine relationships showed similar improvements in both groups, with no significant intergroup differences (Table 4).

Analysis of the molar relationship showed a significant shift toward Class I in both groups from T1 to T2 (*p* < 0.001). In the C-Ela group, the proportion of patients with a Class I molar relationship increased from 0% at T1 to 83.3% at T2, with a corresponding reduction in Class II cases from 77.8% to 5.6%. Similarly, in the TB group, the prevalence of Class I molar relationship rose from 0% to 82.4%, while Class II cases decreased from 82.4% to 11.8%.

For the canine relationship, both groups exhibited a marked improvement, with Class I increasing from 0% at T1 to 88.9% (C-Ela) and 88.2% (TB) at T2 (*p* < 0.001 for each).

At T2, intergroup comparison showed no significant differences in the proportion of patients achieving a Class I molar relationship (C-Ela: 83.3%, TB: 82.4%; *p* = 1.000) or a Class I canine relationship (C-Ela: 88.9%, TB: 88.2%; *p* = 1.000).

Regarding crowding changes (Table 5), the C-Ela group showed a significant improvement in both arches between T1 and T2, with the proportion of subjects presenting normal alignment increasing from 61.1% to 94.4% in the maxilla (*p* = 0.045) and from 38.9% to 88.9% in the mandible (*p* = 0.006). In contrast, no significant changes were observed in the TB group for either the maxilla (58.8% at both T1 and T2; *p* = 1.000) or the mandible (29.4% to 41.2%; *p* = 0.720). Intergroup comparisons at T2 revealed a significantly higher proportion of subjects with normal alignment in the C-Ela group compared to the TB group for both the maxilla (*p* = 0.035) and the mandible (*p* = 0.0089).

Cephalometric analysis (Table 6) revealed that both groups demonstrated a significant reduction in ANB angle from T1 to T2 (C-Ela: *p* = 0.01; TB: *p* = 0.01), indicating sagittal skeletal improvement. SNB increased in both groups (C-Ela: +2.36 mm; TB: +1.45 mm) without a statistically significant change. Vertical skeletal parameters showed a significant increase in Ans-Pns/Go-Gn in the C-Ela group (*p* = 0.04), with no corresponding significant change in the TB group.

Dentoalveolar changes (Table 6) included a significant increase in the interincisal angle for the C-Ela group (C-Ela: *p* < 0.001; TB: *p* = 0.18). Maxillary incisor inclination (U1/S-N) decreased significantly only in the C-Ela group (*p* = 0.01), while lower incisor proclination (L1/Go-Gn) increased significantly in the TB group (*p* = 0.01) but not in the C-Ela group (*p* = 0.08). Moreover, only TB groups showed significant forward positioning of the lower incisors (L1/A-Pog, *p* < 0.001).

Between-group analysis of treatment changes (ΔT2–T1) (Table 6) revealed that the TB group exhibited significantly increases in L1/Go-Gn (*p* < 0.001) and L1/A-Pog (*p* = 0.01), whereas the C-Ela group showed a significantly reduction in U1/S-N (*p* < 0.001) and increases of the interincisal angle (*p* < 0.5). No other skeletal or dentoalveolar variables showed statistically significant differences between groups.

## 4. Discussion

Recent advances in 3D imaging and digital workflow have allowed the full customization of orthodontic devices such as eruption guidance appliances (EGAs), offering a promising alternative in the interceptive treatment of Class II malocclusion [6,27]. In the present study, the dentoskeletal effects of a customized elastodontic device were compared to those of Twin block appliance in growing subjects, using as tools digital models and cephalometric analysis before and after 12 months of treatment.

In line with previous findings [10,28,29,30,31], both appliances produced significant reductions in overjet and overbite within groups, and similar improvements in molar and canine relationships, with no significant intergroup differences in these outcomes.

Cephalometric analysis revealed significant intragroup reductions in ANB angle for both appliances, reflecting sagittal skeletal improvement, but no significant intergroup difference. SNB increased in both, whereas vertical skeletal changes (Ans-Pns/Go-Gn) showed a small but significant increase in the C-Ela group and no change in the TB group; however, these differences between groups were not statistically significant.

Several studies have shown that the TB appliance produces significant skeletal effects, including advancement of the chin, reduction of ANB angle, and posterior repositioning of the maxilla [32,33]. Comparatively, the present study on customized elastodontic appliances revealed similar trends in ANB angle modification and vertical dimension control with an improvement in the maxillo-mandibular divergence after treatment compared to the Twin Block.

A notable difference emerged in dental crowding: the C-Ela group showed significant improvements in both arches, with a higher proportion of subjects achieving normal alignment at T2 compared with the TB group. This may be related to the eruption guidance design and full-arch coverage of the customized device, which facilitates controlled alignment during mixed dentition. In fact, a recent study [6], has shown that custom-made elastodontic appliances demonstrated to be significantly more effective in correcting anterior crowding, the dento-skeletal vertical relation and position of permanent incisor compared to the preformed appliance. This enhanced effectiveness may be further emphasized when compared with functional devices such as the Twin Block, which lack integrated dental eruption guides; the presence of these guides in elastodontic appliances allows simultaneous control of tooth eruption and occlusal development, thereby reducing undesirable dentoalveolar side effects [17].

Concerning incisor inclination L1/Go-Gn and the position of the lower incisors relative to the A-Pog line, the customized elastodontic appliance demonstrated more conservative outcomes, providing better control and reduced anchorage loss of the lower incisors, suggesting better control over dentoalveolar side effects.

Moreover, the upper incisor position (U1/S-N) and interincisal angle improved significantly more in patients treated with the customized elastodontic device compared to those treated with the Twin Block. This difference may be attributed to the 3D digital setup used during the design phase of the customized appliance, which allowed precise incisor control. Additionally, the complete coverage of the incisors within specific niches of the elastodontic device likely enhanced the ability to guide their position more effectively than the labial bow typically used in the Twin Block appliance.

While both devices aim to improve mandibular posture and dental relationships their biomechanical strategies differ. Twin Block utilizes acrylic bite blocks with rigid mandibular positioning, while EGA exerts functional stimuli through softer, elastomeric material and eruption control features.

The TB employs rigid acrylic bite blocks for mandibular advancement, often producing more pronounced skeletal effects during the pubertal growth spurt; the customized elastodontic appliance may offer a more gradual and harmonious adaptation, potentially reducing unwanted dental compensations such as excessive proclination of lower incisors, which is a known limitation of Twin Block therapy [18,32,34].

In terms of vertical control, the present study did not reveal significant intergroup differences in skeletal vertical parameters. However, it should be emphasized that also fort that aspect the biomechanical approaches of the two appliances differ. Twin Block therapy often requires adjustments or trimming of posterior acrylic to promote molar eruption in hypodivergent patients, whereas customized elastodontic appliances incorporate guided eruption zones and digital setup instructions that theoretically allow for targeted anterior intrusion and posterior extrusion. Although these features may represent a clinical advantage, especially in early mixed dentition, when growth potential is still high, and skeletal divergence must be carefully monitored.

This feature may be particularly beneficial in early mixed dentition cases, when growth potential is still high, but skeletal divergence must be carefully monitored.

Overall, both appliances were effective in treating Class II malocclusion during growth, with comparable skeletal outcomes in most variables. The C-Ela showed advantages in crowding resolution and maxillary incisor control, whereas the TB produced proclination of lower incisors. These findings suggest that the C-Ela may be especially suited for early interceptive treatment phases, while the TB remains an established option for more pronounced skeletal correction during peak growth.

### Strengths and Limitations of the Study

A major strength of the present study is the use of a fully digital workflow for appliance design, fabrication, and treatment planning, allowing for highly individualized therapy. The inclusion of both cephalometric and digital model analysis provided a comprehensive evaluation of skeletal and dentoalveolar changes. Furthermore, both groups were well matched in terms of age, growth stage, and initial malocclusion characteristics.

However, some limitations should be acknowledged. The relatively small sample size and the follow-up period limited to 12 months, which may not fully capture long-term skeletal adaptations, especially in growing patients.

Moreover, a limit could be the absence of an untreated control group, which would have allowed for a more precise assessment of the influence of natural growth on the results. However, including untreated growing patients would have required additional radiographic exposure, which was not ethically acceptable in the absence of therapeutic intervention.

Although treatment protocols were standardized, patient compliance with removable appliances—an important determinant of treatment success—can vary and was not objectively monitored. Future research with larger samples, longer follow-up, and randomized designs is needed to confirm and expand upon these findings.

## 5. Conclusions

Both the customized elastodontic appliance and the Twin Block were effective in improving sagittal dental and skeletal relationships in growing patients with Class II malocclusion. Significant improvements in overjet, overbite, and occlusal relationships were observed in both groups, with no statistically significant intergroup differences for most skeletal parameters.

The customized elastodontic appliance was associated with a significantly better control of incisors’ position and crowding resolution in both arches. These differences likely reflect the distinct design and biomechanical characteristics of the two appliances.

The use of customized devices appears to enhance the predictability of treatment outcomes by tailoring biomechanics to individual patient needs, minimizing undesired dental compensations. Future research with larger, randomized controlled trials and extended observation periods is recommended to confirm these results and to assess the long-term stability of dentoskeletal changes.

## Figures and Tables

**Table 1:** The demographic and clinical features of the sample analyzed using independent *t*-tests for age group comparisons and chi-square tests to evaluate proportional differences between genders.

Sample Characteristics	Total (N = 35)	C-Ela Group (N = 18)	TB Group (N = 17)	Significance (*p* Value)
Age (mean ± SD)				
	11.4 ± 0.9	11.1 ± 0.7	11.7 ± 0.5	0.11
Gender/sex? (number of subjects)				
Male	11	6	5	0.13
Female	14	12	12	

Bold: significant difference between groups (*p* value < 0.05).

**Table 2:** Descriptive statistics (mean ± SD) and pre-treatment comparisons between the Customized Elastodontic Group and the Twin Block Group using independent sample *t*-test.

Variables	C-Ela Group (N = 18)	TB Group (N = 17)	*p* Value
	Mean ± SD	Mean ± SD	
SNA (°)	81.58 ± 3.68	82.18 ± 3.04	0.408
SNB (°)	76.72 ± 4.17	77.07 ± 3.75	0.538
ANB (°)	5.05 ± 2.03	4.94 ± 2.21	0.677
S-N/Go-Gn (°)	31.15 ± 4.81	32.24 ± 3.91	0.207
S-N/Ans-Pns (°)	8.97 ± 2.8	9.42 ± 2.33	0.568
Ans-Pns/Go-Gn (°)	22.76 ± 4.41	22.0 ± 3.96	0.323
N-Ans (mm)	44.01 ± 3.76	43.68 ± 3.87	0.766
Ans-Me (mm)	53.91 ± 4.17	54.23 ± 3.57	0.752
Gonial angle°	125.59 ± 7.09	126.0 ± 6.03	0.715
Interincisal angle°	126.26 ± 3.32	125.47 ± 5.16	0.403
U1/S-N (°)	111.2 ± 6.02	110.09 ± 4.4	0.114
L1/Go-Gn (°)	96.0 ± 3.6	95.35 ± 2.3	0.405
L1/A-Pog (mm)	1.7 ± 1.02	1.19 ± 1.22	0.128

Bold: significant difference between groups (*p* value < 0.05).

**Table 3:** Statistical comparison of overjet and overbite (mm) in the Customized Elastodontic (C-Ela) and Twin Block (TB) groups at baseline (T1) and after 1 year of treatment (T2). Paired *t*-tests were used for intragroup analysis; two-tailed independent *t*-tests were used for intergroup comparisons.

	C-Ela Group (N = 18)			TB Group (N = 17)			C-Ela Group	TB Group	C-Ela vs. TB
	T1	T2	*p* Value	T1	T2	*p* Value	ΔT2-T1	ΔT2-T1	*p* Value
Variables	Mean ± SD	Mean ± SD		Mean ± SD	Mean ± SD		Mean ± SD	Mean ± SD	
Overjet (mm)	5.25 ± 1.77	2.48 ± 1.07	**0.000**	4.93 ± 2.12	2.63 ± 1.71	**0.003**	−2.77 ± 2.07	−2.30 ± 2.72	0.57
Overbite (mm)	3.99 ± 1.5	2.2 ± 1.25	**0.001**	3.63 ± 1.85	2.23 ± 1.9	**0.045**	−1.79 ± 1.95	−1.40 ± 2.65	0.62

Bold: significant difference between groups (*p* value < 0.05).

**Table 4:** Changes in molar and canine relationships (Angle’s classification) from pre-treatment (T1) to 1-year post-treatment (T2), with inter-time proportional differences assessed by chi-square analysis.

Angle’s Class	C-Ela Group (N = 18)			TB Group (N = 17)			T2 C-Ela vs. TB
	Molar Relationship			Molar Relationship			Molar Relationship
	T1	T2	*p* Value	T1	T2	*p* Value	*p* Value
Class I	0	15	**0.000**	0	14	**0.000**	
Class II	14	1		14	2		
Class I/II	4	2		3	1		1.00
Total	18	18		17	17		
	Canine relationship			Canine relationship			Canine relationship
	T1	T2	*p* value	T1	T2	*p* value	*p* value
Class I	0	16	**0.000**	0	15	**0.000**	
Class II	14	1		15	1		
Class I/II	4	1		2	1		1.00
Total	18	18		17	17		

Bold: significant difference between groups (*p* value < 0.05).

**Table 5:** Distribution and statistical comparison of anterior crowding severity at T1 and T2, evaluated using chi-square analysis for proportional change.

Crowding Level	C-Ela Group (N = 18)			TB Group (N = 17)			T2 C-Ela vs. TB
Maxilla				Maxilla			Maxilla
	T1	T2	*p* Value	T1	T2	*p* Value	*p* Value
Normal (<3 mm)	11	17	**0.045**	10	10	1.00	
Moderate/severe (>3 mm)	7	1		7	7		**0.035**
Total	18	18		17	17		
Mandible				Mandible			Mandible
	T1	T2	*p* value	T1	T2	*p* value	*p* value
Normal (<3 mm)	7	16	**0.006**	5	7	0.720	
Moderate/severe (>3 mm)	11	2		12	10		**0.0089**
Total	18	18		17	17		

Bold: significant difference between groups (*p* value < 0.05).

**Table 6:** Descriptive statistics (mean ± SD) and comparison of pre- (T1) and post-treatment (T2) values using paired sample *t*-tests for intragroup changes and two-tailed independent *t*-tests for intergroup differences.

Variables	C-Ela Group (N = 18)			TB Group (N = 17)			Δ C-Ela	Δ TB	
	T1 Mean ± SD	T2 Mean ± SD	*p* Value	T1 Mean ± SD	T2 Mean ± SD	*p* Value	T2-T1 Mean ± SD	T2-T1 Mean ± SD	*p* Value
SNA (°)	81.58 ± 3.68	82.24 ± 2.78	0.28	82.18 ± 3.04	82.86 ± 2.71	0.25	0.66 ± 4.61	0.68 ± 4.07	0.93
SNB (°)	76.72 ± 4.17	79.08 ± 3.1	0.10	77.07 ± 3.75	78.52 ± 3.71	0.14	2.36 ± 5.20	1.45 ± 5.28	0.53
ANB (°)	5.05 ± 2.03	3.56 ± 1.65	**0.01**	4.94 ± 2.21	3.16 ± 1.68	**0.01**	−1.49 ± 2.62	−1.78 ± 2.78	0.06
S-N/Go-Gn (°)	31.15 ± 4.81	33.29 ± 4.67	0.10	32.24 ± 3.91	34.23 ± 5.55	0.12	2.14 ± 6.70	1.99 ± 6.79	0.69
S-N/Ans-Pns (°)	8.97 ± 2.8	8.52 ± 2.45	0.69	9.42 ± 2.33	8.92 ± 2.83	0.29	−0.45 ± 3.72	−0.50 ± 3.67	0.81
Ans-Pns/Go-Gn (°)	22.76 ± 4.41	25.38 ± 3.79	**0.04**	22.0 ± 3.96	23.81 ± 4.24	0.08	2.62 ± 5.81	1.81 ± 5.80	**0.02**
N-Ans (mm)	44.01 ± 3.76	44.78 ± 4.16	0.28	43.68 ± 3.87	44.19 ± 4.16	0.36	0.77 ± 5.61	0.51 ± 5.68	0.41
Ans-Me (mm)	53.91 ± 4.17	53.77 ± 4.15	0.46	54.23 ± 3.57	54.26 ± 3.26	0.49	−0.14 ± 5.88	0.03 ± 4.83	0.58
Gonial angle (°)	125.59 ± 7.09	126.26 ± 5.63	0.38	126.0 ± 6.03	125.86 ± 4.35	0.47	0.67 ± 9.05	−0.14 ± 7.44	0.09
Interincisal angle (°)	126.26 ± 3.32	130.36 ± 3.57	**0.00**	125.47 ± 5.16	129.4 ± 3.9	0.18	4.10 ± 4.88	3.93 ± 6.47	**0.04**
U1/S-N (°)	111.2 ± 6.02	106.27 ± 4.72	**0.01**	110.09 ± 4.4	108.29 ± 3.65	0.11	−4.93 ± 7.65	−1.80 ± 5.72	**0.00**
L1/Go-Gn (°)	96.0 ± 3.6	97.7 ± 3.18	0.08	95.35 ± 2.3	99.7 ± 5.78	**0.01**	1.70 ± 4.80	4.35 ± 6.22	**0.00**
L1/A-Pog (mm)	1.7 ± 1.02	2.88 ± 1.13	0.07	1.19 ± 1.22	2.63 ± 1.36	**0.00**	1.18 ± 1.52	1.44 ± 1.83	**0.01**

Bold: significant difference between groups (*p* value < 0.05).

## Data Availability

The data used in the present study are available from the corresponding author on reasonable request.
